# Poor association between dihydropyrimidine dehydrogenase ( *DPYD* ) genotype and fluoropyrimidine‐induced toxicity in an Asian population

**DOI:** 10.1002/cam4.5541

**Published:** 2022-12-16

**Authors:** Masashi Kanai, Takahisa Kawaguchi, Masahito Kotaka, Dai Manaka, Junichi Hasegawa, Akinori Takagane, Yoshinori Munemoto, Takeshi Kato, Tetsuya Eto, Tetsuo Touyama, Takanori Matsui, Katsunori Shinozaki, Shigemi Matsumoto, Tsunekazu Mizushima, Masaki Mori, Junichi Sakamoto, Atsushi Ohtsu, Takayuki Yoshino, Shigetoyo Saji, Fumihiko Matsuda

**Affiliations:** ^1^ Department of Therapeutic Oncology, Graduate School of Medicine Kyoto University Kyoto Japan; ^2^ Center for Genomic Medicine, Graduate School of Medicine Kyoto University Kyoto Japan; ^3^ Gastrointestinal Cancer Center Sano Hospital Kobe Japan; ^4^ Department of Surgery, Gastrointestinal Center Kyoto Katsura Hospital Kyoto Japan; ^5^ Department of Surgery Osaka Rosai Hospital Osaka Japan; ^6^ Department of Surgery Hakodate Goryoukaku Hospital Hakodate Japan; ^7^ Department of Surgery Fukui Ken Saiseikai Hospital Fukui Japan; ^8^ Department of Surgery Kansai Rosai Hospital Amagasaki Japan; ^9^ Department of Gastroenterology Tsuchiura Kyodo General Hospital Ibaraki Japan; ^10^ Department of Surgery Nakagami Hospital Okinawa Japan; ^11^ Department of Gastroenterological Surgery Aichi Cancer Center Aichi Hospital Okazaki Japan; ^12^ Division of Clinical Oncology Hiroshima Prefectural Hospital Hiroshima Japan; ^13^ Department of Real World Data Research and Development Graduate School of Medicine, Kyoto University Kyoto Japan; ^14^ Department of Gastroenterological Surgery Osaka University Graduate School of Medicine Osaka Japan; ^15^ Tokai University School of Medicine Isehara Japan; ^16^ Japanese Foundation for Multidisciplinary Treatment of Cancer Tokyo Japan; ^17^ Tokai Central Hospital Kakamigahara Japan; ^18^ Department of Gastroenterology and Gastrointestinal Oncology National Cancer Center Hospital East Chiba Japan

**Keywords:** Asian population, *DPYD*, fluoropyrimidine, genotyping, toxicity

## Abstract

**Objective:**

Dihydropyrimidine dehydrogenase (*DPYD*) genotype is closely associated with fluoropyrimidine (FP)‐induced toxicities in Caucasian population and European Medicines Agency now recommends *DPYD* genotype‐based FP dosing strategy.

**Patients and Methods:**

The current study aimed to investigate their impact on FP‐related toxicities in an Asian population using genome‐wide association study (GWAS) data set from 1364 patients with colon cancer.

**Results:**

Among 82 variants registered in the Clinical Pharmacogenetics Implementation Consortium, 74 *DPYD* variants were directly genotyped in GWAS cohort; however, only 7 nonsynonymous *DPYD* variants (CPIC variants) were identified and none of the four recurrent *DPYD* variants (DPYD*2A, c.2846A>T, c.1679T>G, c.1236G>A) were included. Seven CPIC variants were investigated for their association with the incidence of FP‐related toxicities; however, none of these variants revealed a significant correlation with FP‐related toxicities.

**Conclusion:**

These data suggested that the *DPYD* genotype registered in CPIC plays a minor role in FP‐related toxicities in an Asian population.

## Graphical abstract

DPYD genotype‐based fluoropyrimidine dosing strategy is recommended in a Caucasian population, In contrast, considering the low prevalence of impaired DPYD variants in an Asian population, universal DPYD screening does not appear to be feasible.

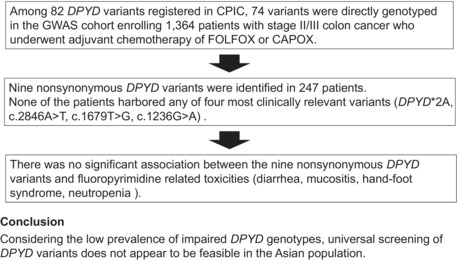

## 1 INTRODUCTION

Fluoropyrimidine (FP) is widely used for a variety of cancers; however, some patients suffer from severe toxicities such as diarrhea, mucositis, hand‐foot syndrome, and neutropenia. Dihydropyrimidine dehydrogenase (*DPYD*) is a key enzyme in FP degradation and approximately 8% of the Caucasian population have reduced *DPYD* genotype and 0.1% having no *DPYD* enzyme activity.^1^, ^2^ Since impaired *DPYD* variants delay FP degradation, patients harboring these variants have the increased risk of developing severe FP‐related toxicities. A recent prospective study demonstrated that FP dose adjustment based on the four recurrent *DPYD* variants (DPYD*2A, c.2846A>T, c.1679T>G, c.1236G>A) could reduce the risk of FP‐related toxicities in the Caucasian population.^2^ A cost analysis revealed that upfront *DPYD* genotyping did not increase the burden of medical expenses and that expected total costs per patient were comparable regardless of whether or not they underwent universal screening.^3^ Based on the aforementioned data, the European Medicines Agency (EMA) has recommended universal *DPYD* genotyping before initiating FP‐based chemotherapy in 2020^4^ and Clinical Pharmacogenetics Implementation Consortium (CPIC) has published 82 *DPYD* variants including 27 variants with impaired enzyme activity^5^; however, it remains unclear whether universal *DPYD* screening and genotype‐based FP dose adjustment are feasible for the Asian population. Therefore, the current study aimed to investigate the prevalence of *DPYD* variants registered in CPIC and its association with FP‐related toxicities using GWAS data from 1364 patients with colon cancer who underwent adjuvant chemotherapy under clinical trials.

## 2 METHODS

### 2.1 Genotyping and clinical data obtained from a genome‐wide association study targeting colon cancer who underwent adjuvant chemotherapy

Genotyping and clinical data obtained from a genome‐wide association study (GWAS) targeting the 1364 patients with colon cancer who underwent adjuvant chemotherapy under clinical trials (JOIN or ACIEVE) were utilized. Among 82 *DPYD* variants registered in the CPIC^5^ (CPIC variants), 74 variants were directly genotyped in GWAS cohort. JOIN was a phase II trial to test the safety of 6 months of modified FOLFOX6 (mFOLFOX6) in Japanese patients with colon cancer.^6^ In contrast, ACHIEVE was a phase III trial planned as a part of phase III trials of the International Duration Evaluation of Adjuvant Chemotherapy (IDEA),^7^ and tested 3 versus 6 months of mFOLFOX6 or capecitabine plus oxaliplatin (CAPOX) adjuvant chemotherapy in patients with colon cancer.^8^, ^9^ Diarrhea/mucositis/hand‐foot syndrome/neutropenia were selected as representative FP‐related toxicities and were graded according to the CTCAE version 3.0 (JOIN) and 4.0 (ACHIVE), respectively. Given those essential elements of diarrhea, mucositis, hand‐foot syndrome, and neutropenia of CTCAE versions 3.0 were equivalent to those of version 4.0, data from both clinical trials were comparable. In both clinical trials, clinical data were collected using an electronic data capture system with regular central monitoring. The primary outcomes of the GWAS were published elsewhere.^10^

### 2.2 Comprehensive search for *DPYD* variants using the Japanese Haplotype Reference Panel database

Nonsynonymous variants of *DPYD* were comprehensively searched using the registry database of the Japanese Haplotype Reference Panel (JHRP, *n* = 3135. https://www.hgvd.genome.med.kyoto‐u.ac.jp/).

### 2.3 Statistical analysis

Associations between the presence of FP‐related toxicities during adjuvant chemotherapy and genotype frequencies of nonsynonymous *DPYD* variants were investigated. *p* values and odds ratios (OR) with 95% confidence intervals (95% CI) were calculated using logistic regression analysis according to the additive genetic model. The significance level was set according to Bonferroni's correction for multiple testing with nine variants (*p* < 0.005). All statistical analyses were performed using R (version 3.2.0; http://www.r‐project.org/).

### 2.4 Ethics

The protocols of GWAS study were approved by the institutional review boards of all participating institutions, and all patients provided written informed consent for the use of genomic and clinical data for research purposes.

## 3 RESULTS

### 3.1 Prevalence of *DPYD* variants registered in CPIC guidelines in GWAS cohort

Among 82 *DPYD* variants registered in CPIC, 74 variants were directly genotyped in GWAS cohort; however, only seven nonsynonymous variants (CPIC variants) were identified and none of the four most clinically relevant *DPYD* variants (DPYD*2A, c.2846A>T, c.1679T>G, c.1236G>A) in the Caucasian population were included. Furthermore, these seven CPIC variants were presumed to have normal function according to the CPIC guidelines (Table 1).

**TABLE 1:** Seven CPIC and two non‐CPIC *DPYD* variants were found in the GWAS cohort

rs number	Nucleotide change/amino acid substitution	CPIC/non‐CPIC	Presumed *DPYD* activity	Minor allele frequency	
				GWAS cohort	JHRP cohort
				(*n* = 1364)	(*n* = 3135)
rs56005131	c.C2303A	CPIC	Normal	0.022	0.025
	p.T768K				
rs1801160	c.G2194A	CPIC	Normal	0.019	0.018
	p.V732I				
rs1801159	c.A1627G	CPIC	Normal	0.28	0.27
	p.I543V				
rs142512579	c.G1294A	CPIC	Normal	0.00036	0
	p.D432N				
rs72549306	c.G1003T	CPIC	Normal	0.00073	0
	p.V335L				
rs2297595	c.A496G	CPIC	Normal	0.02	0.019
	p.M166V				
rs200562975	c.A451G	CPIC	Normal	0.0029	0.0013
	p.N151D				
rs188052243	c.A2678G	Non‐CPIC	Decreased	0.0011	0.0022
	p.N893S				
rs1801265	c.C85T	Non‐CPIC	Unknown	0.04	0.04
	p.R29C				

### 3.2 Comprehensive search for *DPYD* variants using JHRP database

Comprehensive search for *DPYD* variants using the JHRP database identified 53 exonic (1 frameshift insertion, 39 nonsynonymous, and 13 synonymous) variants (Table S1). Eleven variants were overlapped with those registered in CPIC guidelines. However, the four recurrent *DPYD* variants were not registered in the JHRP database comprising 3135 Japanese subjects. Among the 42 variants not registered in CPIC guidelines, two variants were directly genotyped in GWAS cohort and were identified (non‐CPIC variants). One variant (rs188052243) had been reported to have decreased enzymatic activity in vitro (Table 1).^11^

### 3.3 Association between seven CPIC and two non‐CPIC *DPYD* variants and FP‐related toxicities

The prevalence of the seven CPIC and two non‐CPIC *DPYD* variants were compared between those who had grade 0 and grade ≥2 or 3 diarrhea, mucositis, hand‐foot syndrome, and neutropenia; however, no significant associations were observed (Table 2).

**TABLE 2:** Association between the presence of each nonsynonymous *DPYD* variants and fluoropyrimidine‐related toxicities in the GWAS cohort (*n* = 1364)

rs number	Toxicities	Minor allele frequency in patients with indicated toxicity (%)	Odds ratio	95% Confidence interval	*p*‐value
rs56005131	Diarrhea	4.1	1.8	0.62–5.2	0.26
G>T	Mucositis	1.7	0.79	0.18–3.4	0.74
	Neutropenia	2.2	0.99	0.50–1.9	0.98
	Hand‐foot syndrome	1.3	0.36	0.049–2.7	0.49
rs1801160	Diarrhea	4.1	2.2	0.75–6.4	0.14
C>T	Mucositis	0	N/A	N/A	N/A
	Neutropenia	1.8	0.92	0.44–1.8	0.82
	Hand‐foot syndrome	2.7	1.6	0.16–14.6	0.36
rs1801159	Diarrhea	25.5	0.84	0.53–1.3	0.46
T>C	Mucositis	38.3	1.5	1.0–2.2	0.024
	Neutropenia	29.4	1.0	0.84–1.2	0.7
	Hand‐foot syndrome	28.0	0.98	0.49–1.9	0.92
rs142512579	Diarrhea	0	N/A	N/A	N/A
C>T	Mucositis	0	N/A	N/A	N/A
	Neutropenia	0	N/A	N/A	N/A
	Hand‐foot syndrome	0	N/A	N/A	N/A
rs72549306	Diarrhea	1	21.1	1.2–341.5	0.032
C>A	Mucositis	0.89	19.4	1.2–315.7	0.036
	Neutropenia	0.15	N/A	N/A	N/A
	Hand‐foot syndrome	0	N/A	N/A	N/A
rs2297595	Diarrhea	5.1	3.4	1.2–9.0	0.015
T>C	Mucositis	2.6	1.3	0.40–4.5	0.61
	Neutropenia	2.2	1.1	0.56–2.2	0.71
	Hand‐foot syndrome	2.7	1.3	0.20–9.3	0.59
rs200562975	Diarrhea	0	N/A	N/A	N/A
T>C	Mucositis	0	N/A	N/A	N/A
	Neutropenia	0.47	5.1	0.53–49.4	0.15
	Hand‐foot syndrome	0	N/A	N/A	N/A
rs188052243	Diarrhea	0	N/A	N/A	N/A
T>C	Mucositis	0	N/A	N/A	N/A
	Neutropenia	0	N/A	N/A	N/A
	Hand‐foot syndrome	0	N/A	N/A	N/A
rs1801265	Diarrhea	5.1	1.6	0.64–4.4	0.3
A>G	Mucositis	2.3	1.19	0.25–2.7	0.76
	Neutropenia	2.0	0.53	0.28–1.0	0.054
	Hand‐foot syndrome	1.3	0.4	0.049–2.7	0.21

*Note*: As for diarrhea and neutropenia, patients who developed grade 3 or 4 events were counted. As for mucositis and hand‐foot syndrome, patients who developed grade 2 or higher events were counted. “N/A” meant that odds ratio could not be estimated due to having zero cases. Uncorrected *p*‐values were shown.

## 4 DISCUSSION

Since 2020, EMA has recommended universal *DPYD* genotyping before initiating FP‐based chemotherapy.^4^ In this study, we aimed to investigate the feasibility of *DPYD* genotype‐based FP dosing strategy in an Asian population. The four most clinically relevant *DPYD* variants (DPYD*2A, c.2846A>T, c.1679T>G, c.1236G>A) in the Caucasian population were not found in both GWAS cohort (*n* = 1364) and the JHRP database (*n* = 3135), which were in line with the previous study searching for *DPYD* variants in 341 Japanese subjects.^12^ Comprehensive search for nonsynonymous *DPYD* variants using the JHRP database identified 42 *DPYD* variants which were not published in the CPIC guideline; however, most of them were rare and median minor allele frequency (MAF) was 0.0006 (range 0.0001–0.28, Table S1). Only two variants were directly genotyped and were identified in GWAS cohort. Notably, no significant association had been found between seven CPIC and two non‐CPIC *DPYD* variants identified in the GWAS cohort and FP‐related toxicities. Two patients harboring presumed impaired *DPYD* enzymatic activity (rs188052243) did not develop severe FP‐related toxicities.

Thus, the prevalence of *DPYD* genotype registered in CPIC guideline is quite low and plays a minor role in FP‐related toxicities in an Asian population. In contrast, incidence of FP‐related toxicities in Japanese patients were comparable or higher than those in Caucasian patients (Table S2). For example, the incidence of grade 3 or higher neutropenia observed in this cohort (23.2%) was significantly higher compared to that in the IDEA trial excluding ACHIEVE cohort (8.2%) (Table S2). This suggests that the development of FP‐related toxicities in the Asian population could be attributed to factors other than the *DPYD* variants registered in CPIC guideline.

We also evaluated the association between other hematological toxicities (leucopenia, anemia, thrombocytopenia, and febrile neutropenia) and nine nonsynonymous *DPYD* variants in our GWAS cohort; however, no significant associations were found (data not shown).

The current study has some limitations. First, although data regarding FP‐related toxicities were collected in large prospective clinical trials, their actual relationship with FP was unclear given that patients received a combination chemotherapy of FP and oxaliplatin. Second, most of rare *DPYD* variants identified in Japanese subjects with presumed impaired enzymatic activity^11^, ^13^ were not directly genotyped in GWAS cohort. Therefore, we could not exclude the possibility of rare *DPYD* variants were involved in FP‐related toxicities in the Asian population.

In summary, the current study suggested that *DPYD* genotypes registered in CPIC guidelines play a minor role in FP‐related toxicities in the Asian population and that universal *DPYD* screening does not appear to be feasible.

## AUTHOR CONTRIBUTIONS

**Masashi Kanai:** Conceptualization (lead); data curation (lead); formal analysis (lead); investigation (equal); project administration (lead); writing – original draft (lead). **Takahisa Kawaguchi:** Data curation (lead); formal analysis (lead); methodology (lead); validation (lead); writing – original draft (lead). **Masahito Kotaka:** Investigation (equal). **Dai Manaka:** Investigation (equal). **Junichi Hasegawa:** Investigation (equal). **Akinori Takagane:** Investigation (equal). **Yoshinori Munemoto:** Investigation (equal). **Takeshi Kato:** Investigation (equal). **Tetsuya Eto:** Investigation (equal). **Tetsuo Touyama:** Investigation (equal). **Takanori Matsui:** Investigation (equal). **Shinozaki Katsunori:** Investigation (equal). **Shigemi Matsumoto:** Conceptualization (supporting); investigation (equal). **Tsunekazu Mizushima:** Investigation (equal). **Masaki Mori:** Project administration (supporting). **Junichi Sakamoto:** Project administration (supporting); writing – review and editing (lead). **Atsushi Ohtsu:** Project administration (supporting). **Takayuki Yoshino:** Conceptualization (lead); project administration (lead); supervision (lead). **shigetoyo saji:** Project administration (lead); supervision (lead). **Fumihiko Matsuda:** Conceptualization (lead); project administration (lead); supervision (lead); writing – review and editing (equal).

## FUNDING INFORMATION

This study was funded by Yakult Honsha Co., Ltd. Grant number is not applicable.

## CONFLICT OF INTEREST

Masashi Kanai reports stock from Therabiopharma Inc., honoraria from Chugai Pharmaceutical Co., Ltd., and research grant from Molecular Health. Masahito Kotaka reports honoraria from Chugai Pharmaceutical Co., Ltd., and Yakult Honsha Co., Ltd. Takeshi Kato reports honoraria from Chugai Pharmaceutical Co., Ltd., Takeda Pharmaceutical Company Limited, Taiho Pharmaceutical Co., Ltd, ONO Pharmaceutical Co., and Eli Lilly Japan K.K. Katsunori Shinozaki reports honoraria from Chugai Pharmaceutical Co., Ltd., Mochida Pharmaceutical Co. Ltd., Taiho Pharmaceutical Co., Ltd., Eli Lilly Japan K.K., Daiichi Sankyo Co. Ltd., Bayer Yakuhin, Ltd., Ono Pharmaceutical Co., Ltd., Eisai Co., Ltd., SHIONOGI & Co., Ltd., Merck Biopharma Co., Ltd., Nippon Kayaku Co., Ltd., and Kyowa Kirin Co., Ltd. Takayuki Yoshino reports honoraria from Taiho Pharmaceutical Co., Ltd., Chugai Pharmaceutical Co., Ltd., Eli Lilly Japan K.K., Merck Biopharma Co., Ltd., Bayer Yakuhin, Ltd., Ono Pharmaceutical Co., and MSD K.K., and research funds from MSD K.K. (Inst), Daiichi Sankyo Co., Ltd. (Inst), Ono Pharmaceutical Co., Ltd (Inst), Taiho Pharmaceutical Co., Ltd (Inst), Amgen K.K. (Inst), Sanofi K.K. (Inst), Pfizer Japan Inc, (Inst), Genomedia (Inst), Sysmex Corporation (Inst), Nippon Boehringer Ingelheim Co., Ltd (Inst), PAREXEL International Inc. (Inst), Chugai Pharma Co., Ltd (Inst). Fumihiko Matsuda reports research grant from Yakult Honsha Co., Ltd.

## ETHICS STATEMENT

The protocols of GWAS study were approved by the institutional review boards of all participating institutions, and all patients provided written informed consent for the use of genomic and clinical data for research purposes.

## Supporting information


Table S1.

Table S2.

## Data Availability

The data that support the findings of this study are available on request from the corresponding author. The data are not publicly available due to privacy or ethical restrictions.

## References

[1] Meulendijks D , Henricks LM , Sonke GS , et al. Clinical relevance of DPYD variants c.1679T>G, c.1236G>A/HapB3, and c.1601G>A as predictors of severe fluoropyrimidine‐associated toxicity: a systematic review and meta‐analysis of individual patient data. Lancet Oncol. 2015;16:1639‐1650. 26603945 10.1016/S1470-2045(15)00286-7

[2] Henricks LM , Lunenburg C , de Man FM , et al. DPYD genotype‐guided dose individualisation of fluoropyrimidine therapy in patients with cancer: a prospective safety analysis. Lancet Oncol. 2018;19:1459‐1467. 30348537 10.1016/S1470-2045(18)30686-7

[3] Henricks LM , Lunenburg C , de Man FM , et al. A cost analysis of upfront DPYD genotype‐guided dose individualisation in fluoropyrimidine‐based anticancer therapy. Eur J Cancer. 2019;107:60‐67. 30544060 10.1016/j.ejca.2018.11.010

[4] European Medicines Agency . Fluorouracil and fluorouracil related substances Article 31 referral – EMA recommendations on DPD testing prior to treatment with fluorouracil, capecitabine, tegafur and flucytosine; EMA/367286/2020.

[5] Amstutz U , Henricks LM , Offer SM , et al. Clinical Pharmacogenetics Implementation Consortium (CPIC) guideline for Dihydropyrimidine dehydrogenase genotype and Fluoropyrimidine dosing: 2017 update. Clin Pharmacol Ther. 2018;103(2):210‐216. 29152729 10.1002/cpt.911 PMC5760397

[6] Yoshino T , Kotaka M , Shinozaki K , et al. JOIN trial: treatment outcome and recovery status of peripheral sensory neuropathy during a 3‐year follow‐up in patients receiving modified FOLFOX6 as adjuvant treatment for stage II/III colon cancer. Cancer Chemother Pharmacol. 2019;84:1269‐1277. 31549217 10.1007/s00280-019-03957-5 PMC6820589

[7] Grothey A , Sobrero AF , Shields AF , et al. Duration of adjuvant chemotherapy for stage III colon cancer. N Engl J Med. 2018;378:1177‐1188. 29590544 10.1056/NEJMoa1713709 PMC6426127

[8] Yoshino T , Yamanaka T , Oki E , et al. Efficacy and long‐term peripheral sensory neuropathy of 3 vs 6 months of Oxaliplatin‐based adjuvant chemotherapy for colon cancer: the ACHIEVE phase 3 randomized clinical trial. JAMA Oncol. 2019;5(11):1574‐1581. 31513248 10.1001/jamaoncol.2019.2572 PMC6743062

[9] Yoshino T , Oki E , Misumi T , et al. Final analysis of 3 versus 6 months of adjuvant Oxaliplatin and Fluoropyrimidine‐based therapy in patients with stage III colon cancer: the randomized phase III ACHIEVE trial. J Clin Oncol. 2022;40:3419‐3429. 35512259 10.1200/JCO.21.02628

[10] Kanai M , Kawaguchi T , Kotaka M , et al. Large‐scale prospective genome‐wide association study of oxaliplatin in stage II/III colon cancer and neuropathy. Ann Oncol. 2021;32:1434‐1441. 34391895 10.1016/j.annonc.2021.08.1745

[11] Hishinuma E , Narita Y , Saito S , et al. Functional characterization of 21 allelic variants of Dihydropyrimidine dehydrogenase identified in 1070 Japanese individuals. Drug Metab Dispos. 2018;46:1083‐1090. 29769267 10.1124/dmd.118.081737

[12] Maekawa K , Saeki M , Saito Y , et al. Genetic variations and haplotype structures of the DPYD gene encoding dihydropyrimidine dehydrogenase in Japanese and their ethnic differences. J Hum Genet. 2007;52:804‐819. 17828463 10.1007/s10038-007-0186-6

[13] Hishinuma E , Narita Y , Obuchi K , et al. Importance of rare DPYD genetic polymorphisms for 5‐fluorouracil therapy in the Japanese population. Front Pharmacol. 2022;13:930470. 35784703 10.3389/fphar.2022.930470 PMC9242541

